# Corneal vesicles accumulate collagen VI associated with tissue remodeling in apolipoprotein a-I deficiency: a case report

**DOI:** 10.1186/s12886-017-0404-8

**Published:** 2017-02-08

**Authors:** Hiroyuki Namba, Mari Narumi, Shinji Susa, Rintaro Ohe, Takeo Kato, Mitsunori Yamakawa, Hidetoshi Yamashita

**Affiliations:** 1 Department of Ophthalmology and Visual Sciences, Yamagata University Faculty of Medicine, 2-2-2 Iidanishi, 9909585 Yamagata City, Yamagata Japan; 2 Department of Neurology, Hematology, Metabolism, Endocrinology and Diabetology, Yamagata University Faculty of Medicine, Yamagata City, Yamagata Japan; 3 Department of Diagnostic Pathology, Yamagata University Faculty of Medicine, Yamagata City, Yamagata Japan

**Keywords:** Case report, Apolipoprotein a-I deficiency, Collagen VI, Confocal microscopy, Corneal opacity, Immunohistochemistry

## Abstract

**Background:**

Apo A-I deficiency clinically shows low serum levels of HDL cholesterol and corneal opacity at a young age. Histopathological evaluations of affected corneas are not enough, and the mechanism of corneal opacity is still unclear.

**Case presentation:**

A 61-year-old woman suffered from blurred vision with a corneal opacity. She had significantly reduced serum levels of high-density lipoprotein cholesterol and Apo A-I, stenosis of the coronary arteries, and ischemic heart failure. On genetic examination, a homozygous mutation of Apo A-I_Tsukuba_ was identified. Histopathological examination of the corneal button after PKP showed numerous vesicles in the corneal stroma, which were more prominent in the deep stroma than in the shallow stroma. Collagen VI was observed in some of those vesicles.

**Conclusion:**

We experienced a rare case of corneal opacity due to Apo A-I deficiency. Our histopathological findings indicated that structural changes in corneal collagen fibrils contribute to the formation of stromal vesicles.

## Background

Apo A-I is an essential component of HDL. Its deficiency is clinically detected by low serum levels of HDL cholesterol. Although different gene mutations of Apo A-I are reported [1–4], the typical clinical presentation includes corneal opacity, xanthoma, and ischemic heart disease at a young age. Histopathological evaluations of affected corneas are not enough, and the mechanism of corneal opacity is still unknown. In the present study, we report a part of the mechanism associated with corneal opacity in a case of genetic Apo A-I deficiency.

## Case presentation

In 2012, a 61-year-old woman with blurred vision presented with corneal opacity of the left eye. Her visual acuity was 20/40 OS and 20/20 OD. She had been diagnosed with HDL deficiency at the age of 46 years, 1997. but she had been off treatment for about 10 years. She had undergone PKP of the right eye for the same symptoms when she was 58 years old, 2009.

The patient was the fourth of 8 siblings. A brother died in an accident and a sister died in childhood. None of her family members had been diagnosed with HDL deficiency, and there was no family history of consanguineous marriage. On physical examination, she had bilateral xanthomas of the eyelids and focal yellow skin discolorations in the cubital fossae of both forearms, but there was no tonsillar hypertrophy or Achilles tendon xanthoma.

**Fig. 1:**
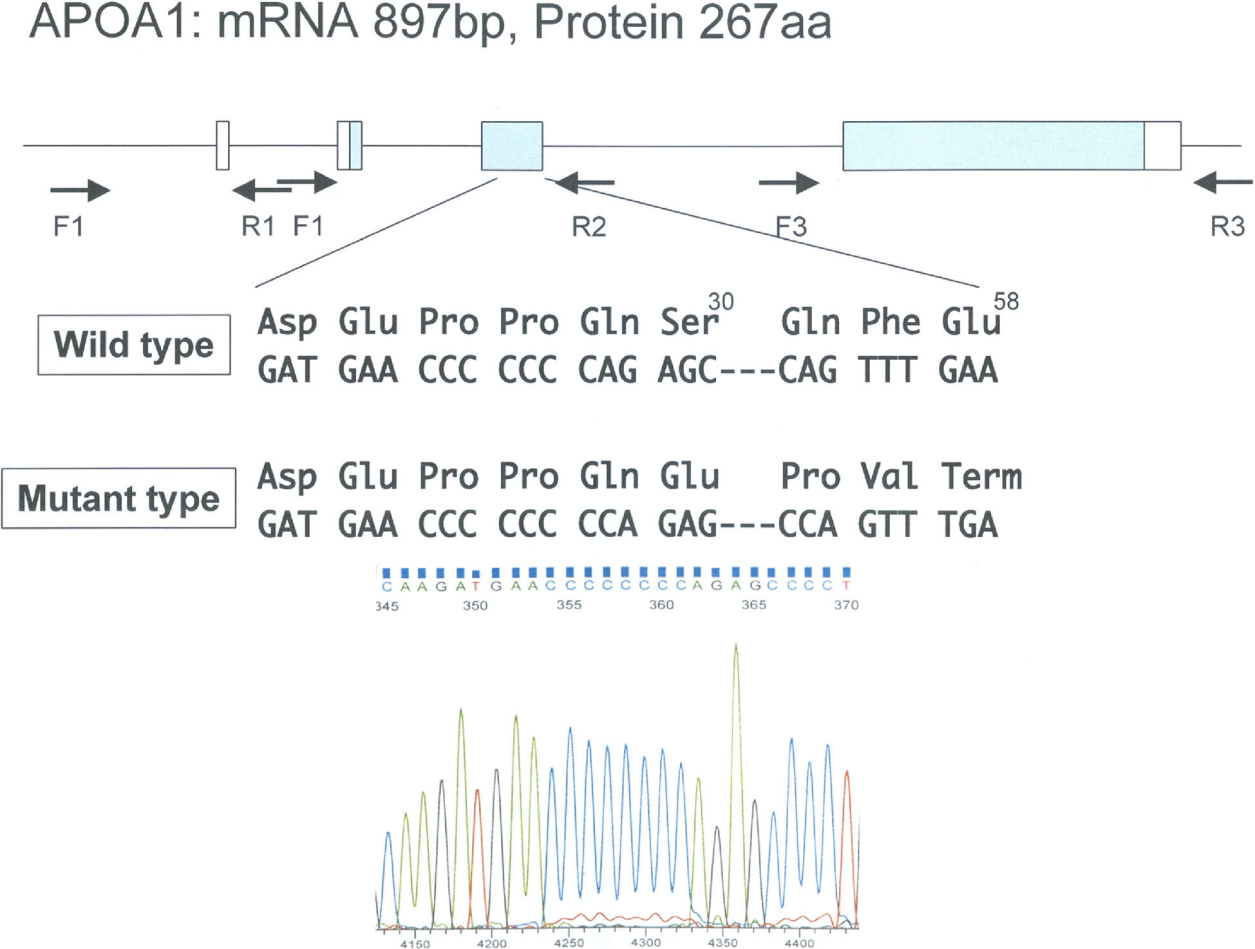
The result of genetic evaluation. A single-nucleotide insertion of cytosine altered the reading frame from codon 5 and produces a premature stop codon at codon 34

Blood tests showed an extremely low HDL-cholesterol level (6 mg/dL) with undetectable Apo A-I (<5 mg/dL). Levels of Apo B and Apo E were slightly elevated (109 mg/dL and 5.8 mg/dL, respectively). Serum triglyceride, total cholesterol, low-density lipoprotein cholesterol, and lecithin-cholesterol acyltransferase levels were within the normal limits. A genetic evaluation was performed as previously described [4], and showed that she was homozygous for the Apo A-I_Tsukuba_ mutation, which is a single-nucleotide insertion of cytosine that alters the reading frame from codon 5 and produces a premature stop codon at codon 34 (Fig. 1). Thus, this patient was diagnosed with genetic Apo A-I deficiency. Electrocardiogram, echocardiogram, and myocardial scintigraphy showed significant ischemic heart failure and angiography showed severe stenosis of the coronary arteries. Consequently, she underwent percutaneous coronary intervention and coronary artery bypass grafting.

**Fig. 2:**
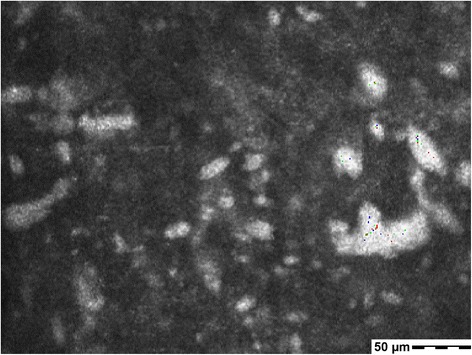
Confocal microscopy in the corneal stroma. High-intensity vesicles, 10 to 100 μm in diameter, were observed

On slit-lamp examination, there was a diffuse corneal stromal opacity with a lucid zone in the left eye. Confocal microscopy (Heidelberg Retinal Tomograph Rostock Cornea Module, Heidelberg Engineering, Heidelberg, Germany) revealed high-intensity vesicles (10–100 μm in diameter) in the corneal stroma (Fig. 2). In 2013, PKP was performed for the left eye, and the corneal button tissues were examined histopathologically and immunohistochemically.

### Histopathological and immunohistochemical analysis of the corneal button

**Fig. 3:**
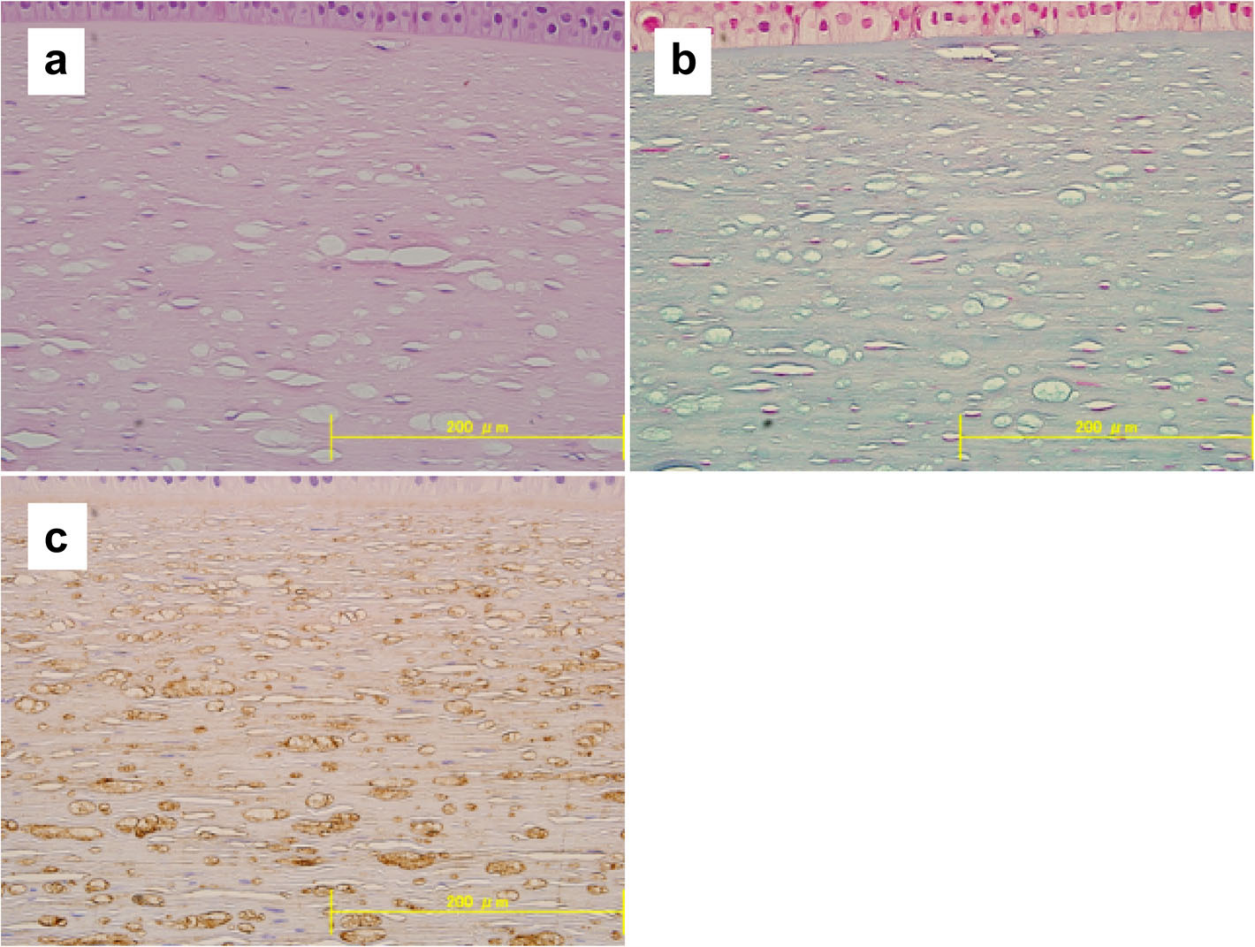
Histopathology and immunohistochemistry of the surgically excised corneal button. **a** Histopathology of the central cornea. There were many small vesicles (10 to 100 μm in diameter) the corneal stroma. The vesicles were much more numerous in the deep stroma than in the upper stroma (hematoxylin and eosin staining; magnification × 40). **b** Histopathology of the central cornea. Accumulations in the vesicles were alcian-blue positive (alcian-blue staining; magnification × 40). **c** Collagen VI immunostaining of the central cornea detected collagen VI in some of the vesicles (magnification × 40)

The cornea showed normal thickness and there was no significant abnormality of the epithelium, Bowman’s layer, or the endothelium. There were numerous small vesicles in the corneal stroma (Fig. 3a), which were of similar size to the vesicles observed with confocal microscopy. The vesicles were more prominent in the deep stroma than in the shallow stroma. Accumulations in the vesicles were Congo-red negative and alcian-blue positive (Fig. 3b). Sudan III and Sudan black staining could not confirm the presence of cholesterol ester in the vesicles. On immunohistochemical analysis, collagen VI [clone: anti-hCL (VI), mouse IgG1,κ; 1:500; KYOWA PHARMA CHEMICAL CO., LTD., Toyama, Japan] was observed in some vesicles (Fig. 3c).

Since the PKP, the patient has been on regular medication with pitavastatin and tocopherol nicotinate and has had a normal lipid profile except for low serum HDL-cholesterol levels. In 2015, her current visual acuity is 20/25 OS and 20/20 OD and both corneal grafts have maintained good transparency.

## Discussion

Our patient demonstrated clinical findings typical to Apo A-I deficiency. Histopathologically, vesicles in the corneal stroma were significantly larger (10–100 μm in diameter) than those described in the report by Osakabe and colleagues (200 nm–2 μm in diameter) [5]. Apo A-I mutations, including Apo A-I_Iowa_, are known to induce amyloidosis [6]. However, our patient did not have any accumulation of amyloid in the corneal stroma. It is possible that we failed to detect cholesterol ester in the vesicles by Sudan black staining because we embedded the corneal button in paraffin. It was also regretful that the right cornea had not been examined immunohistochemically.

Collagen VI is a major extracellular matrix protein, bridging cells to the surrounding connective tissue and organizing the three-dimensional tissue architecture. The mutations in the genes encoding Collagen VI chains result in several forms of inherited myopathies, decrease of stiffness in cartilage [7]. It is also distributed in corneal stroma, and reported the change of its expression level during wound healing period [8–10]. 

## Conclusions

To our knowledge, ours is the first report of a patient with confirmed mucoid degeneration and accumulations of collagen VI in the stromal vesicles. Our results suggest that structural changes in corneal collagen fibrils contribute to the formation of stromal vesicles.

## Abbreviations

Apo: Apolipoprotein
HDL: High-density lipoprotein
OD: Oculus Dexter
OS: Oculus Sinister
PKP: Penetrating keratoplasty
